# Supplementary figures and images for: The Host Immune Response to Scedosporium/Lomentospora
Source: J Fungi (Basel). 2021 Jan 22;7(2):75. doi: 10.3390/jof7020075 (PMC7912657; doi:10.3390/jof7020075)

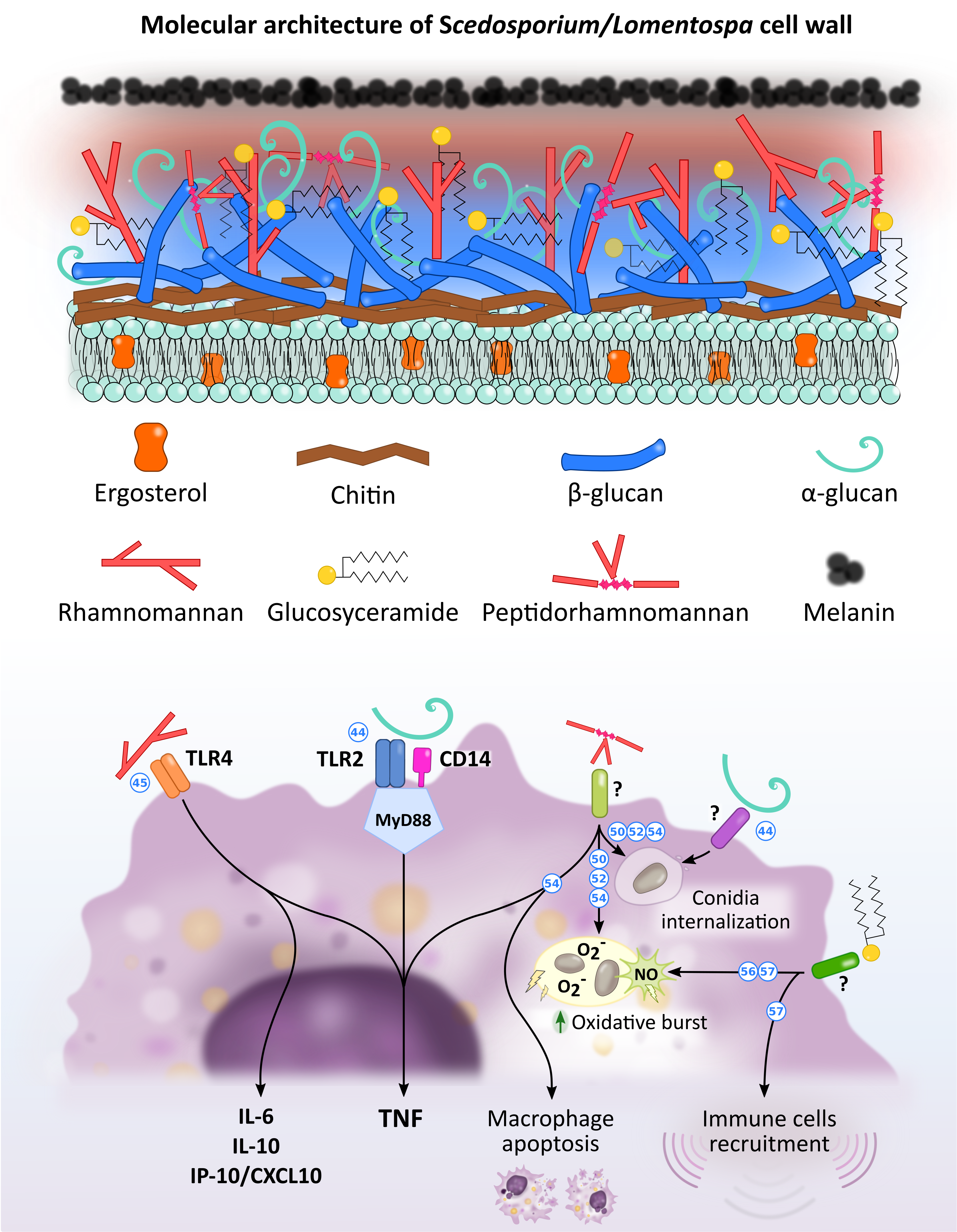

Supplement: Supplementary file 1 [file jof-07-00075-s001.zip › Supplementary Material/Figure S1.tif]

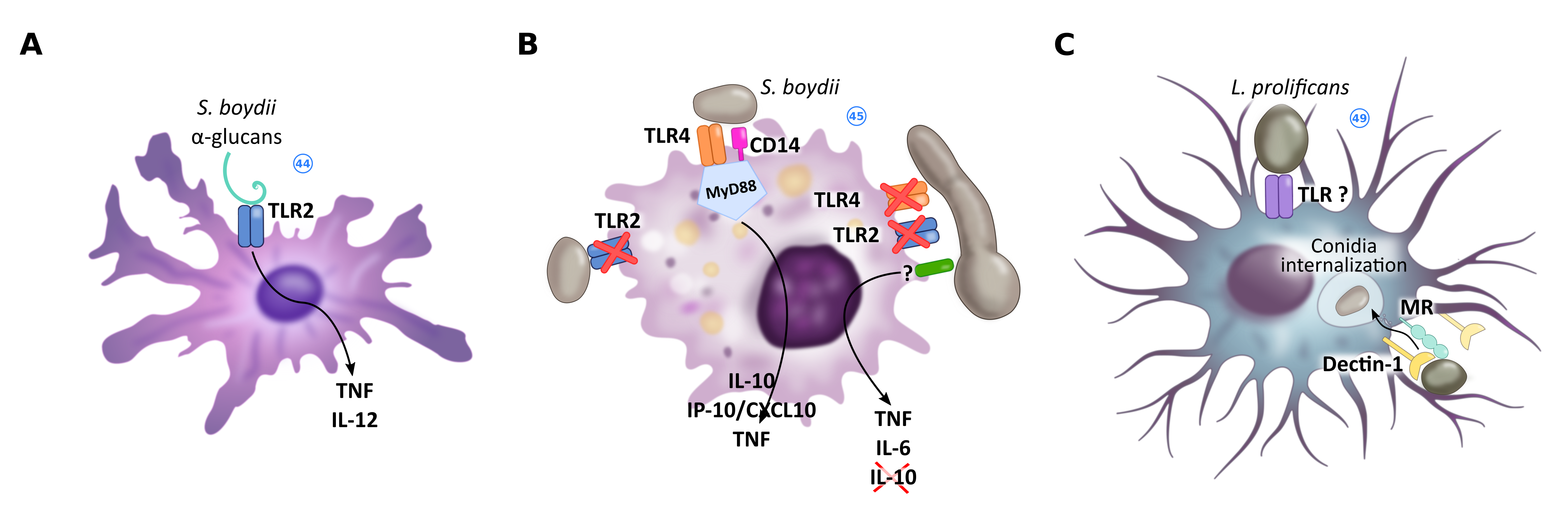

Supplement: Supplementary file 1 [file jof-07-00075-s001.zip › Supplementary Material/Figure S2.tif]

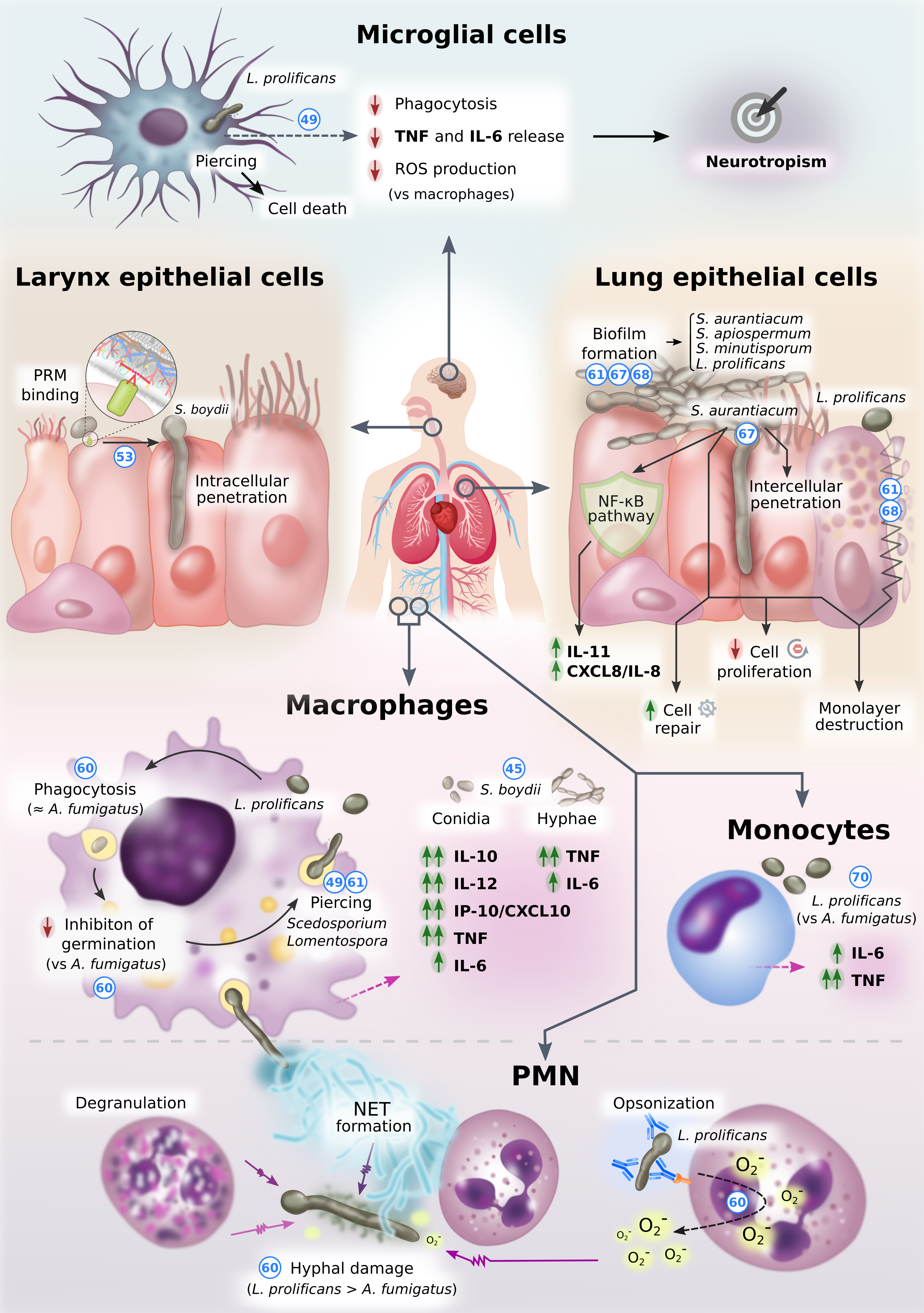

Supplement: Supplementary file 1 [file jof-07-00075-s001.zip › Supplementary Material/Figure S3.tif]
